# Evaluating the value of a 3D printed model for hands-on training of gynecological pelvic examination

**DOI:** 10.1186/s41205-022-00149-5

**Published:** 2022-07-06

**Authors:** Matthias Kiesel, Inga Beyers, Adam Kalisz, Achim Wöckel, Anne Quenzer, Tanja Schlaiß, Christine Wulff, Joachim Diessner

**Affiliations:** 1 University Hospital Würzburg department of Gynecology, Josef-Schneider-Str. 4, 97080 Würzburg, Germany; 2 Institute of Electric Power Systems (IfES), Leibniz Universität Hannover, Appelstraße 9A, 30167 Hannover, Germany; 3 Department of Electrical, Electronic and Communication Engineering, Information Technology (LIKE), Friedrich-Alexander-Universität Erlangen-Nürnberg, Am Wolfsmantel 33, Erlangen, Germany

**Keywords:** Gynecology, Pelvic examination, Pelvic palpation, 3D printing, Teaching, Visualization, Education, Pelvisio®

## Abstract

**Background:**

Simulation in the field of gynecological pelvic examination with educational purposes holds great potential. In the current manuscript we evaluate a 3D printed model of the female pelvis, which improves practical teaching of the gynecological pelvic examination for medical staff.

**Methods:**

We evaluated the benefit of a 3D printed model of the female pelvis (Pelvisio®) as part of a seminar (“skills training”) for teaching gynecological examination to medical students. Each student was randomly assigned to Group A or B by picking a ticket from a box. Group A underwent the skills training without the 3D printed model. Group B experienced the same seminar with integration of the model. Both groups evaluated the seminar by answering five questions on Likert scales (1–10, 1 = “very little” or “very poor”, 10 equals “very much” or “very good”). Additionally, both groups answered three multiple-choice questions concerning pelvic anatomy (Question 6 to 8). Finally, Group B evaluated the 3D printed model with ten questions (Question 9 to 18, Likert scales, 1–10).

**Results:**

Two of five questions concerning the students’ satisfaction with the seminar and their gained knowledge showed statistically significant better ratings in Group B (6.7 vs. 8.2 points and 8.1 vs. 8.9 points (*p* < 0.001 and *p* < 0.009). The other three questions showed no statistically significant differences between the traditional teaching setting vs. the 3D printed model (*p* < 0.411, *p* < 0.344 and *p* < 0.215, respectively). The overall mean score of Question 1 to 5 showed 8.4 points for Group B and 7.8 points for Group A (*p* < 0.001). All three multiple-choice questions, asking about female pelvic anatomy, were answered more often correctly by Group B (p < 0.001, *p* < 0.008 and p < 0.001, respectively). The mean score from the answers to Questions 9 to 18, only answered by Group B, showed a mean of 8.6 points, indicating, that the students approved of the model.

**Conclusion:**

The presented 3D printed model Pelvisio® improves the education of female pelvic anatomy and examination for medical students. Hence, training this pivotal examination can be supported by a custom designed anatomical model tailored for interactive and explorative learning.

## Background and aim

Ample training of female pelvic examination is work- and time intensive and yet of crucial significance in the field of Gynecology. We experience numerous medical students lacking adequate comprehension and feeling great insecurity. Furthermore, everyday clinical workload and the intimate nature of this exam pose further challenges for sufficient education of female examination including rectovaginal palpation. In addition, many students feel insecure performing genital examination on actual patients [1–5]. This leads to the necessity of high-quality simulation. Until now, this need has only been partly met, with few studies covering the field of simulating gynecological examination [4–7]. Yet, there is data showing that 3D printed teaching models can indeed be of aid for education of medical staff [8–13].

**Fig. 1:**
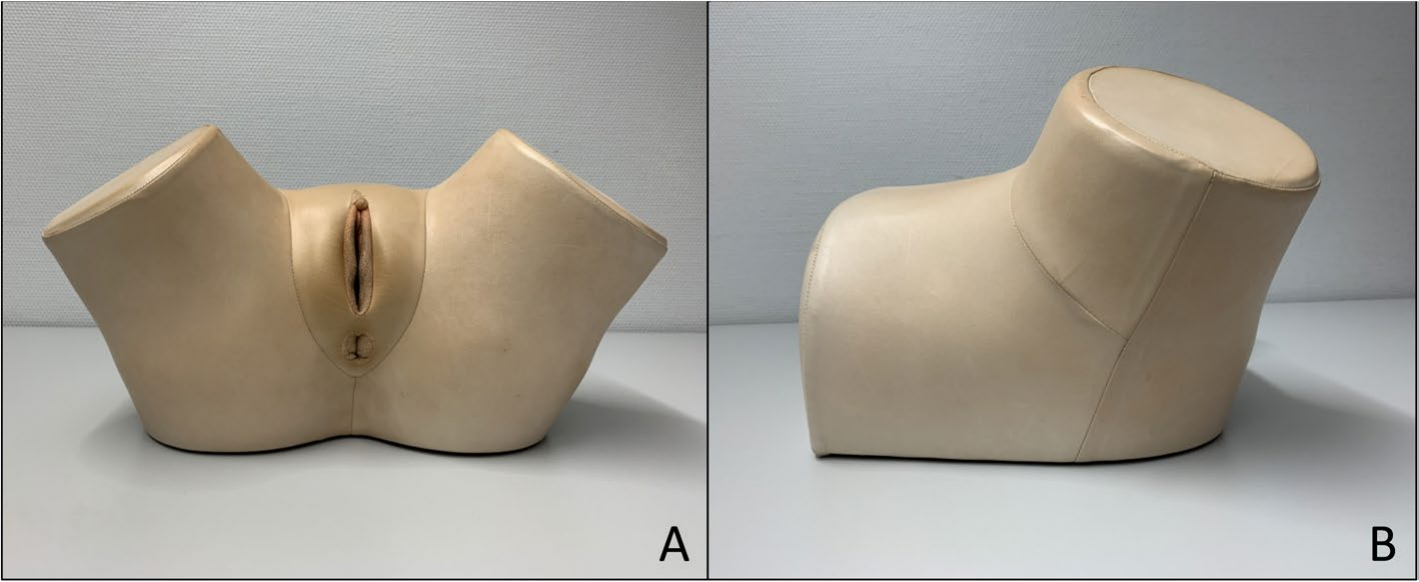
Palpation-based model of the company *Schultes medacta GmbH & Co Lehrmodelle KG*

Medical students in their fifth to sixth year experience standard curricular education for gynecological examination at the Department of Gynecology of the University Hospital Würzburg. This “skills training” takes place once for every student, lasts 1.5 hours and consists of verbal explanations together with the presentation of 2D images by using Microsoft PowerPoint. Basic information about physiology, anatomy and the core steps of gynecological examination are taught in form of a lecture. This freshly gained theoretical knowledge is then actively trained. This takes place in the hands-on part of the skills training. Traditionally, only one type of model has been used, namely commercial models of the company *Schultes medacta GmbH & Co Lehrmodelle KG*. These models do not display any internal anatomy, but solely offer the possibility of palpation. We experience many students struggling to imagine and to comprehend the concerning anatomical structures by only palpating, i.e. feeling the models’ fabric without sufficient visualization. A picture of this palpation-based model is depicted in Fig. 1. Consequently, we integrated the novel 3D printed model to bridge the gap between haptic and visual sensation. In this work, we evaluate the model’s effect on our students’ education as well as their satisfaction with the model and the skills training.

## Methods

### The 3D printed model

**Fig. 2:**
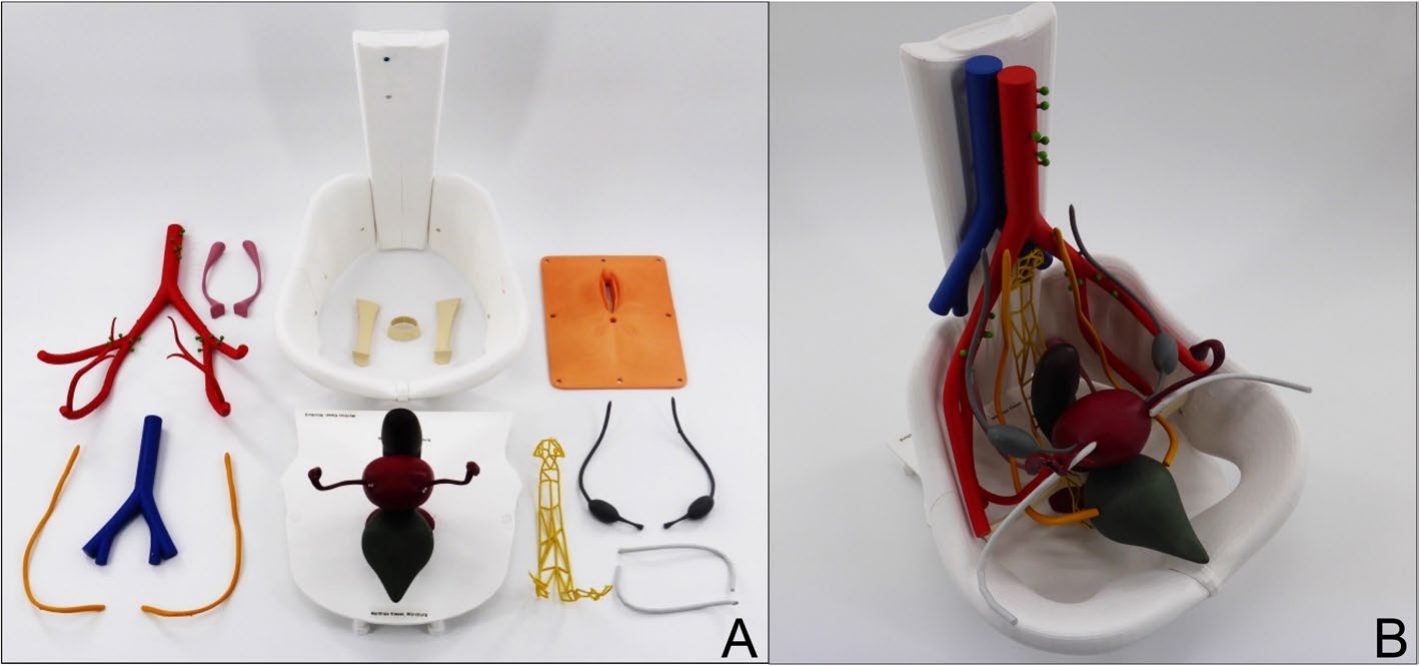
3D printed model Pelvisio® for training of pelvic examination. **A** Depiction of the model disassembled into single parts. **B** Depiction of entirely assembled model

We utilized the program Blender, version 2.93, to create a virtual and simplified model depicting key parts of the female pelvis. The illustrations and anatomical data are based on clinical experience as well as the information in the book *PROMETHEUS Lernatlas der Anatomie, 2nd edition* from *Georg Thieme Verlag KG* and *Taschenbuch Anatomie*, 1st edition from *Elsevier GmbH*. The decision, which structures should be shown, was motivated by our clinical experience. Our major aspiration was to visualize pivotal structures, in order to support medical students’ comprehension and memorization. Using the 3D printing techniques Material Extrusion together with Stereolithography (SLA), we produced the 26 single parts of the model, which can be attached manually. To enable the assembly by students, the single organs and structures have different colors as well as fastening mechanisms which facilitate the connection of the single parts. Laying the model on the spine, i.e. tilting it by 90°, it simulates the real position during gynecological examination. A depiction of the model disassembled into single parts, as well as entirely assembled can be seen in Fig. 2. A detailed description of the development and manufacturing process as well as the single parts of Pelvisio® is found in our previous work {DOI 10.1186/s41205-022-00139-7, manuscript TDPM-D-21-00013R2 accepted in 3D printing in medicine}.

### Incorporating the 3D printed model into the skills training

The novel 3D printed model is presented to the students in its dismantled state. Every student group is asked to assemble the model. Consequently, the theoretical knowledge of the prior lecture can be applied practically. During this period of the skills training, the physician assists the students to assemble the model, corrects mistakes and answers questions. When every group has assembled their 3D printed model, the commercial models of the company *Schultes medacta GmbH & Co Lehrmodelle KG* were used. Except for vulva, vagina and cervix, these models’ internal anatomy can only be examined by palpation. The students then begin to train the inspection of the female genitalia, the use of different specula, the performance of PAP-smear and the bimanual, rectovaginal palpation. While doing so, each group can continually correlate and compare their findings between the 3D printed and the commercial models. The skills training ends with the students answering an evaluation-form.

In order to evaluate the effect of the 3D printed model, 84 students, randomized into two different groups, took part in our skills training from October to November 2020 as single-center study.

Each student-group experiencing the skills training consisted of 6–8 students and was randomly assigned to Group A or B by picking a ticket from a box. Group A (38 students) received training without the 3D printed model. These students listened to the theoretical introduction at the beginning of the skills training and then directly commenced the practical training by working with the commercial models of the company *Schultes medacta GmbH & Co Lehrmodelle KG*. They did not see the 3D printed model. Group B (46 students) additionally experienced the 3D printed model as described. The size of Group A and B vary, as student groups differed between 6 to 8 participants and as single students had to cancel or postpone their participation in the skills training. All trainings were conducted by the same physician with five years of experience in practical examination, in order to exclude inter-teacher-variability.

All students were older than 18 years and gave their consent in voluntarily participating in this work. All information gained was anonymized. A certificate of non-objection was obtained from the Ethics Committee of the University Hospital Würzburg (application number 2019050201).

Both groups assessed the skills training with an evaluation-form of which the first part consisted of Question 1 to 5 asking for a personal assessment of the skills training. These questions were to be answered on a Likert scale (1–10, 1 = “very little” or “very poor”, 10 equals “very much” or “very good”). Question 4 was inverted, asking about the insecurity students felt when thinking about their first real pelvic examination. Higher scores indicate greater insecurity. Furthermore, Group A and Group B both answered three identical multiple-choice questions (Question 6 to 8) focusing on pelvic anatomy, pathophysiology and examination, in order to test the knowledge they had gained during the skills training. These three questions were rated as right / wrong, depending on the answers the students gave. Moreover, Group B answered a special evaluation-form specifically assessing the 3D printed model and its benefits during the skills training. These questions 9 to 18 were also answered on a Likert scale (1–10) with higher scores indicating higher satisfaction with the model or agreement with the question. The questions can be found in the Appendix of this work.

Group A and B were compared with regards to their subjective evaluation of the skills training with or without the 3D printed model (Question 1–5) together with their objectively obtained knowledge (Question 6–8). In order to prevent any disadvantage for Group A, the 3D printed model was demonstrated to every student and everyone was allowed to work and learn with the 3D printed model after finishing the evaluation of the skills training.

### Statistics

Statistical analysis was done by SPSS Version 25. The significance level was 0.050. To compare whether the difference between Group A and B was statistically significant concerning Question 1 to 5 (Likert scale 1–10), the Mann-Whitney-U-Test was used. Fisher’s exact test was utilized to evaluate if the difference in Question 6 to 8 (multiple-choice question, answered right / wrong) between Group A and B was statistically significant.

## Results

The 3D printed model in this work can be used for teaching gynecological pelvic examination to medical staff. It helps visualize anatomical structures and serves the improved understanding of the female pelvic anatomy.

**Fig. 3:**
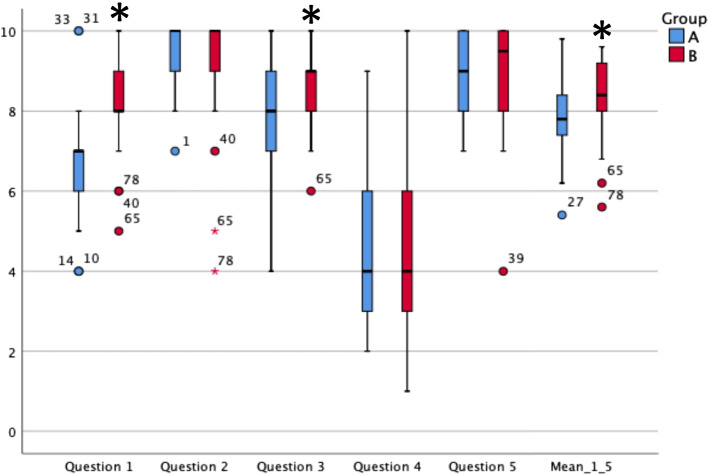
Boxplots visualizing the answers to Question 1–5 as well as the mean score of all answers (Likert scales 1–10, 1 = “very little” or “very poor”, 10 equals “very much” or “very good”). The boxes indicate the interquartile range and the black bar in the middle of each box shows the median. The whiskers stand for minimum and maximum point scores. The dots and asterisks depict the outliers. * indicate statistically significant differences between the concerning boxplots of Group A and Group B. Blue bars and dots refer to Group A (training without 3D printed model) and red bars and dots refer to Group B (training with 3D printed model)

When comparing the scores in Question 1 to 5 between Group A and B, it becomes evident, that Group B (training with 3D printed model) rated the skills training better than Group A. This difference was statistically significant between the scores in Question 1 (How well informed do you feel about the overall anatomy of the female pelvis?): Group A rated this question with a mean of 6.7 points. Group B rated with 8.2 points (*p* < 0.001). Question 3 (Did you understand the anatomical structures and relationships crucial for gynecological examination?) scored 8.1 points in Group A and 8.9 points in Group B (*p* < 0.009). For the scores of Question 2 (Did you understand the single steps of the explained examination?), 4 (How insecure do you feel about performing a gynecological examination of a real patient in the future?) and 5 (Please asses the quality of the skills training concerning its learning success for you personally) the differences showed no statistical significance (*p* < 0.411, *p* < 0.344 and *p* < 0.215, respectively). However, when viewing the mean score of Question 1 to 5 together, a statistically significant difference between both groups can be seen, indicating that Group B rated the skills training better than Group A (Group A: 7.8 points, Group B: 8.4 points, *p* < 0.001). Boxplots further visualize these findings in Fig. 3.

Question 6 to 8 consisted of multiple-choice questions with each 5 statements. The students were asked to choose the correct statements from the list. All questions contained more than one correct choice. If all correct statements were marked, the answer was classified as correct. If a wrong statement was marked or if not all correct statements were recognized as such, the answer was classified as incorrect. Question 6 (Please mark the anatomical structure(s), which play(s) an important role during regular gynecological examination.), Question 7 (Please mark the anatomical structure(s), which play(s) an important role during gynecological examination for local staging of cervical cancer.) and Question 8 (Please mark the anatomical structure(s), which is / are part of the connective tissue holding the uterus.) were answered correctly more often by Group B than by Group A. This difference was statistically significant (*p* < 0.001 for Question 6, *p* < 0.008 for Question 7 and p < 0.001 for Question 8).

**Fig. 4:**
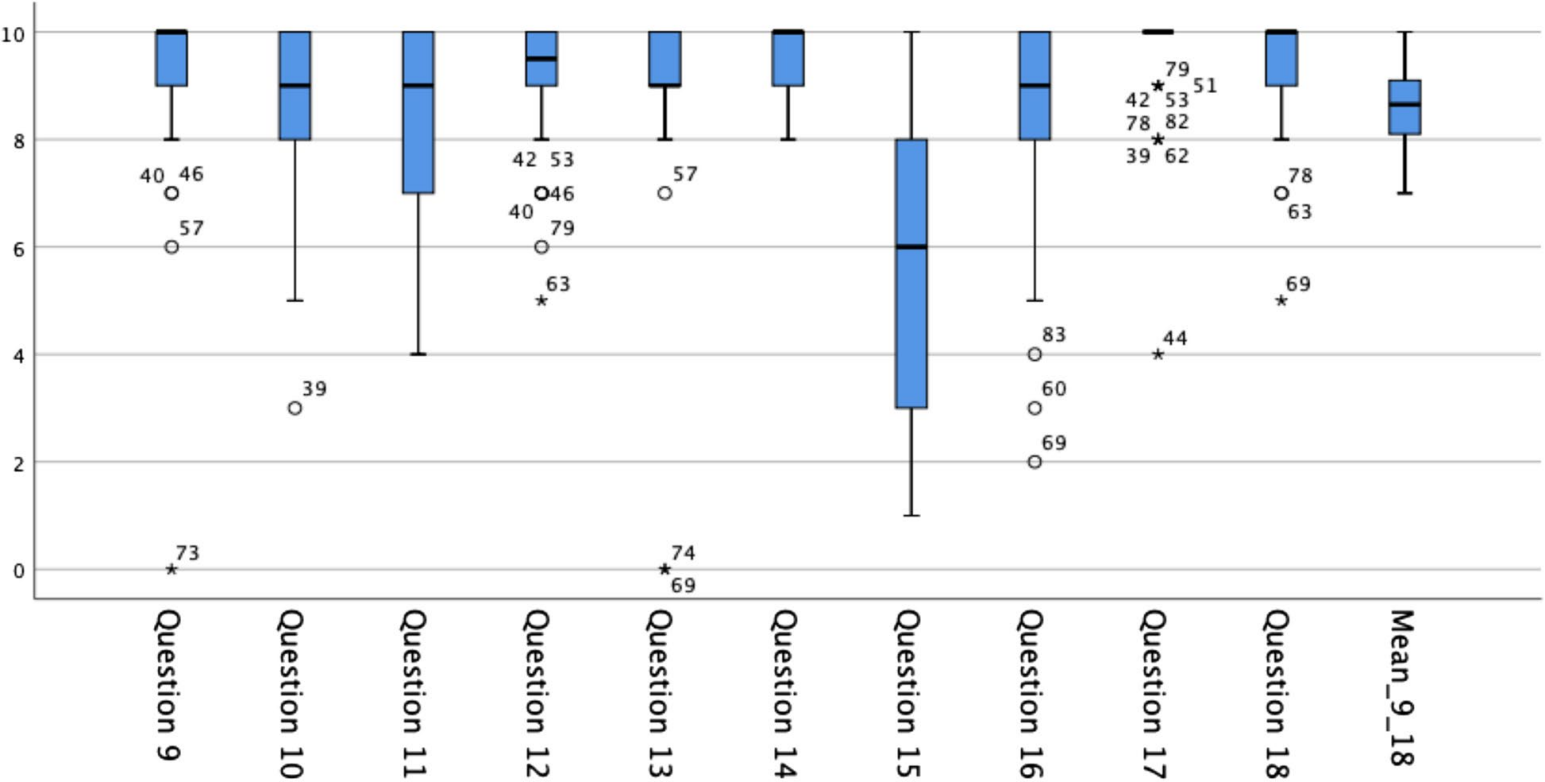
Boxplots visualizing the answers to Question 9 to 18 (only Group B, training with 3D printed model) as well as the mean score of all answers (Likert scales 1–10, 1 = “very little” or “very poor”, 10 equals “very much” or “very good”). The boxes indicate the interquartile range and the black bar in the middle of each box shows the median. The whiskers stand for minimum and maximum point scores. The dots and asterisks depict the outliers

Questions 9 to 18 were only answered by Group B. They contained Likert scales (1–10, 1 = “very little” or “very poor”, 10 equals “very much” or “very good”), with which the students could rate the 3D printed model, its usefulness in the skills training and further aspects in the field of simulation and additive manufacturing for medical training and education. The 46 students of Group B rated Question 9 (How helpful was the 3D model for your understanding of the anatomy?) and Question 10 (How helpful were the different colors of the single parts of the 3D model for your understanding of the anatomy?) with a mean of each 9.0 points. Question 11 (How helpful was the possibility to remove single parts of the 3D model for your understanding?) was rated with a mean of 8.2 points. The students gave a mean of 9.1 points to Question 12 (Was the possibility to use the 3D model in two different positions (standing upright and tilted to 90 degrees) useful?). Question 13 (Did the model altogether improve the quality of the skills training?) scored a mean of 8.9 points. A mean of 9.7 points was given to Question 14 (Would you recommend the use of plastic 3D models, such as the one you experienced today, for medical students’ education?) and a mean of 5.5 points was given to Question 15 (Would you recommend the use of solely virtual 3D models for medical students’ education?). Question 16 (Would you recommend the use of augmented reality for medical students’ education?) scored a mean of 8.2 points. Moreover, Question 17 (Would you approve of models showing certain medical conditions / illnesses for medical students’ education? (E.g. cervical / ovarian cancer, myoma, endometriosis, etc.)?) received a mean of 9.6 points. Finally, a mean of 9.3 points was used for rating Question 18 (Would you approve of models, which can be used by medical students for simulating gynecological operations via laparoscopy / laparotomy / vaginal operation?). When creating a mean score from the answers to Question 9 to 18, it becomes evident, that the students gave an overall rating of 8.6 points. This is further visualized in Fig. 4.

## Discussion

The printed model of the pelvis can support health care professionals and patients to better comprehend important anatomical structures of the female pelvis. When combining it with models for pure palpation, its educational potential can be increased. The emphasis on those aspects, which are crucial for rectovaginal palpation, facilitates understanding and keeping an overview. In addition, the active involvement of the students by assembling the model themselves, enables further improved learning results.

### Evaluation of the 3D printed model

The model’s potential is shown firstly by the subjective assessment of the skills training. Question 1 was rated by Group A with a mean of 6.7 and by Group B with 8.2 points (*p* < 0.001). Question 3 scored 8.1 points in Group A and 8.9 points in Group B (*p* < 0.009). Adding to this, the mean of all points scored from Question 1 to 5 also favored Group B (Group B: 8.4 points, Group A: 7.8 points, *p* < 0.001). This highlights, that those students, who were able to learn with the 3D printed model, felt better educated about female pelvic anatomy and examination than those without the model.

Secondly, this subjective opinion of the students is affirmed, when viewing Question 6 to 8. These multiple-choice questions aimed at objectively testing the students’ knowledge and understanding of the anatomic coherences they just learned about. Group B answered all three multiple-choice questions more often correctly than Group A with statistical significance concerning this difference (p < 0.001 for Question 6, *p* < 0.008 for Question 7 and p < 0.001 for Question 8). This can be seen as evidence, that the 3D printed model indeed increases the learning results of medical students.

Thirdly, by asking the students of Group B about the 3D printed model and novel approaches towards teaching, interesting findings could be made. When being asked how helpful the model itself and its differently colored parts were for their understanding of the concerning anatomy (Question 9 and 10), the students rated these two questions with a mean of each 9.0 points, emphasizing their support of the 3D printed model. This fact fits well to the answers of Group B to Question 1 (How well informed do you feel about the overall anatomy of the female pelvis) and Question 3 (Did you understand the anatomical structures and relationships crucial for gynecological examination?), which both favored teaching with the 3D printed model. The interactive aspects of Pelvisio® were also appreciated by the students: Question 11 (How helpful was the possibility to remove single parts of the 3D model for your understanding?) and Question 12 (Was the possibility to use the 3D model in two different positions (standing upright and tilted to 90 degrees) useful?) both scored above 8 points, indicating, that activating the students and giving them the possibility of participating in hands-on learning was perceived positively. Hence, it appears congruent that Question 13 (Did the model altogether improve the quality of the skills training?) scored a mean of 8.9 points and Question 14 (Would you recommend the use of plastic 3D models, such as the one you experienced today, for medical students’ education?) was rated with a mean of 9.7 points. This underlines the 3D printed model’s potential and showcases the importance of practical learning and participation, especially, when taking into account the students’ rather divided opinion towards the use of solely virtual 3D models for medical students’ education (Question 15, rated with a mean of 5.5 points). Yet, when combined with hands-on-aspects, again allowing activation during education, such as with augmented reality, the idea receives the students’ appreciation, as can been seen with Question 16 (Would you recommend the use of augmented reality for medical students’ education?) scoring a mean of 8.2 points.

When discussing possible future aims, the students of Group B showed, that they were interested in further projects beyond the 3D printed model, such as phantoms showing certain medical conditions or illnesses, e.g. cervical or ovarian cancer, myoma or endometriosis, by rating Question 17 with a mean of 9.6 points. In addition, taking the idea of realistic simulation in medical education one step further, the students clearly approved of the idea of models, which could be used for simulating gynecological operations (mean of 9.3 point for Question 18). Thus, the demand for future projects for high quality model-based simulation is highlighted.

### Existing data and limitations

There have been studies emphasizing the potential of simulation with models for gynecological education. Yet, they are often relatively outdated, such as the work of Takestraw et al. from 1985 [14], Johnson et al. from 1975 [15], Nelson et al. from 1978 [16] and Holzmann et al. from 1977 [17]. It is to be assumed that technical possibilities were different than today, making a direct comparison to current studies difficult.

Many of the promising modern studies, such as from Wånggren et al. rely on so called Gynaecology Teaching Associates (GTAs) [7, 18–20]. These are professionally prepared patients, who interact with the examiner during the examination, providing him or her with feedback. Although this undoubtedly is a valuable addition to the teaching of practical skills, GTAs are expensive. Additionally, the organization of a GTA-based training is more difficult and the cooperation with such trained patients is only common in selected countries. Effective teaching relies on methods, which are swiftly implemented and not too resource-intensive. Consequently, this leads to GTAs being difficult to introduce in many healthcare systems.

The data of Pugh et. al. indicates that models of the female pelvis equipped with pressure sensors could be a valuable alternative. They were superior to pure lectures and to training with regular models [7, 21, 22]. Nevertheless, only little data is available and, here as well, potentially higher costs must be noticed together with a more resource intensive production.

There are no studies, which focus on the evaluation of models constructed for primary visualization in addition to using them together with models for palpation, in order to teach pelvic examination. Moreover, we could find no study examining the use of a 3D printed female pelvic model, which was to be assembled by the students as part of the teaching process.

A more detailed comparison to the current scientific work, together with a display of the expenses for equipping an institution with the described 3D printed model and an overlook about the increased accessibility as well as the logistical and financial advantages of 3D printing in the context of teaching medical staff is described in our previous work {DOI 10.1186/s41205-022-00139-7, manuscript TDPM-D-21-00013R2 accepted in 3D printing in medicine}.

Limitations to this study must be stated concerning the evaluation-form of Question 4 (How insecure do you feel about performing a gynecological examination of a real patient in the future?). Whereas all questions were to be rated according to the Likert scale 1–10 (1 = “very little” or “very poor”, 10 equals “very much” or “very good”), a high score in Question 4 is to be seen as negative. This inconsistency reduced the quality of our methods and should be corrected in future studies. Moreover, three multiple-choice questions must be criticized as too few, in order to adequately compare the gained knowledge and comprehension between the two groups. The organization of the entire training was planned in order to compare Group A to Group B. Consequently, we could provide the identical settings for both groups, with the same physician for teaching and the same time-frame and content for the lecture as well as the practical training part. Nevertheless, using these settings to perform a direct comparison between two models could have produced more valuable data than the simple comparison of a group being taught with the 3D printed model and a group without any additional input for visualization. Although case numbers in this work are higher than in most previous studies, further projects with more participants, possibly organized as multi-center studies, should be carried out.

## Conclusion

The model Pelvisio® offers a promising tool for bridging the gap between visualization and palpation during the training of gynecologic examination. Its focus on the core anatomical structures and the active assembly from ground up add to a deeper understanding and increased retention of the gained information. Hence, a contribution can be made to educating future health care providers in this important field of clinical diagnostics. The possibility of reducing the parts of the model to those structures necessary for a specific demonstration and explanation also opens the option of using the model for e.g. preoperative patient education. Until now, there is a lack of studies examining modern aspects of simulation for the field of Gynecology. Further research in these fields should be conducted.

## Data Availability

The datasets used and analysed during the current study are available from the corresponding author on reasonable request.

## Abbreviations

AR: Augmented Reality
GTA: Gynaecology Teaching Associates
SLA: Stereolithography
3D: Three Dimensional
